# Age, Growth and Spatial Distribution of the Life Stages of the Shortfin Mako, *Isurus oxyrinchus* (Rafinesque, 1810) Caught in the Western and Central Atlantic

**DOI:** 10.1371/journal.pone.0153062

**Published:** 2016-04-06

**Authors:** Rodrigo R. Barreto, Wialla K. T. de Farias, Humber Andrade, Francisco M. Santana, Rosangela Lessa

**Affiliations:** 1 Laboratório de Dinâmica de Populações Marinhas (DIMAR), Departamento de Pesca e Aquicultura, Universidade Federal Rural de Pernambuco, Recife, PE, Brazil; 2 Laboratório de Modelagem Estatística (MOE), Departamento de Pesca e Aquicultura, Universidade Federal Rural de Pernambuco, Recife, PE, Brazil; Virginia Commonwealth University, UNITED STATES

## Abstract

The shortfin mako (*Isurus oxyrinchus*) is a highly migratory pelagic shark that preferentially inhabits oceanic regions in practically all oceans. The wide distribution range of this species renders it susceptible to coastal and oceanic fishing operations. The International Union for Conservation of Nature (IUCN) and the International Commission for the Conservation of Atlantic Tunas (ICCAT) consider this species to be highly vulnerable, especially due to its biological parameters, which are different from those of other sharks that occupy the same niche (e.g., *Prionace glauca*). Consequently, considerable declines in abundance have been detected over various parts of its range, most of which are linked to oceanic longline fishing. The species has conflicting life history parameters in studies conducted in the last 30 years, especially with regard to age and growth. The main discrepancies regard the interpretation of the periodicity of the deposition of band pairs (BPs) on vertebrae and the possibility of ontogenetic variations in growth. Shortfin mako sharks (n = 1325) were sampled by onboard observers of the Brazilian chartered pelagic longline fleet based in northeast Brazil from 2005 to 2011. Lengths were 79 to 250 and 73 to 296 cm (fork length, FL) for males and females, respectively, with a statistically significant difference in size between sexes and differences in the proportion of individuals in each size class. The onboard observers collected a subsample of vertebrae (n = 467), only 234 of which were suitable for analyses. Reliability between readings was satisfactory. However, it was not possible to validate periodicity in the formation of age bands in the sample. Thus, the von Bertalanffy growth function was used to calculate growth rates for the species through the interpretation of BPs in different scenarios: one BP per year (s1), two BPs per year (s2) and two BPs per year until five years of life (s3). Growth parameters varied for both females (Linf = 309.7[s3] to 441.6[s1]; k = 0.04[s1] to 0.13[s3]; t0 = -7.08[s1] and -3.27[s3]) and males (Linf = 291.5[s3] to 340.2[s1]; k = 0.04[s1] to 0.13[s3]; t0 = -7.08[s1] and -3.27[s3]). To advance the understanding of the use of habitat, the first analysis of the spatial distribution of the life stages of the shortfin mako sharks caught by commercial longline fishing operations in the South Atlantic was performed. The findings indicate that the portion of the population exploited by the fleets is predominantly juvenile and future actions should take the following issues into account: improvements in the efficiency of data collection procedures, the reestablishment of the onboard observer program, emergency investments in studies that can contribute to a better understanding of habitat use and life history theory.

## Introduction

The shortfin mako shark (*Isurus oxyrinchus*) is a highly migratory pelagic shark [1] that preferentially inhabits oceanic regions in practically all oceans from about 50° N to 50° S and even up to 60° in some regions (i.e., the Northeast Atlantic) [2–4]. This species uses a heat-exchanging circulatory system to keep its internal temperature above that of the environment and is able to perform large-scale migrations, making it one of the most active and powerful fishes and probably the fastest shark [1,5–7].

The wide distribution range of the shortfin mako renders it susceptible to coastal and oceanic fisheries. This species is highly representative in industrial longline fishing operations that target tunas and billfishes [2, 3] and ranks second after the blue shark (*Prionace glauca*) among frequent species of shark [8–10]. Unlike the majority of sharks (for which only the fins are prized), there is also a high commercial demand for the meat of the shortfin mako, especially in countries of the European Union, such as Spain [9–11]. Thus, this shark attracts more economic interest than other sharks, including the blue shark, which has substantially higher catch rates [3, 9, 10]. Moreover, the shortfin mako is one of the most prized species in recreational fishing due to its physical strength, with numerous fishing tournaments occurring around the world [2, 12].

Sharks of the family Lamnidae are predicted to be less resilient than other shark species in situations of high mortality rates due to their high longevity, late maturity and extremely low fertility and productivity rates [2, 13–15]. Declines in abundance have been detected in various parts of the distribution range of the shortfin mako, most of which are attributed to longline fisheries [3, 10, 16–19]. In 2009, the International Union for Conservation of Nature (IUCN) ranked the species as vulnerable (VU) based on inferred declines worldwide, inadequate management and continuous fishing pressure [7].

A major concern, however, is that catches have not been properly recorded and studies on the life history of the shortfin mako (biological parameters) report conflicting results [2,7]. Regarding reproduction, considerable differences in size at sexual maturity have been reported in studies conducted in the North Atlantic and Pacific Oceans [2, 7, 20–26]. Information on age and growth is even more conflicting [2,7, 27–37].

Some authors assume an annual pattern of band pair (BP) deposition [21, 25, 29–36], while others assume a biennial pattern [27, 28, 37]. Both patterns have been validated, specifically for two BPs per year until only the first five years of life [37]. Accurate age determination is important to stock assessments and the management of fisheries, as it is necessary to calculate growth rates, longevity, mortality rates and age at first maturity [38–40]. These parameters are fundamental to stock assessments conducted by fishery management agencies, such as the Food and Agriculture Organization (FAO) and the International Commission for Conservation of Atlantic Tunas (ICCAT), as well as conservation organizations, such as the IUCN and the Convention on International Trade in Endangered Species of Wild Fauna and Flora (CITES).

In the Atlantic Ocean, industrial longline fisheries account for approximately 25% of reported shark catches on the global scale [9]. Studies report that catch rates for this species have declined more the 50% in both hemispheres in recent decades [10, 16, 17]. The ecological risk assessment approach [14, 15] recently adopted by the ICCAT in data-poor situations, which is based on biological productivity and susceptibility, has demonstrated that the shortfin mako is likely to be the most vulnerable shark to longline fisheries in the Atlantic [14, 15].

Despite contrasting life history parameters for this species worldwide, only one study particularly on age and growth, with vertebrae collected off the coast of southern Brazil, is available for the species in the South Atlantic [36]. Thus, the aim of the present study was to provide further information on the age and growth of the shortfin mako in the area, with specimens sampled more to the north and center than the study cited. The von Bertalanffy growth function (*VBGF*) was used to calculate growth rates for the species through the interpretation of band pairs (BPs) on vertebrae based using different scenarios of periodicity. To advance the understanding of habitat use, we also offer the first analysis on the spatial distribution of the life stages of the shortfin mako sharks caught by commercial longline fisheries in the South Atlantic. Protocols for biological sampling in the Brazilian Exclusive Economic Zone were conducted in compliance with Brazilian regulations for wildlife research and approved by the Chico Mendes Biodiversity Conservation Institute (ICMBio) of the Brazilian Ministry of the Environment (permit no. 49663–1).

## Methods

### Sample

**Fig 1:**
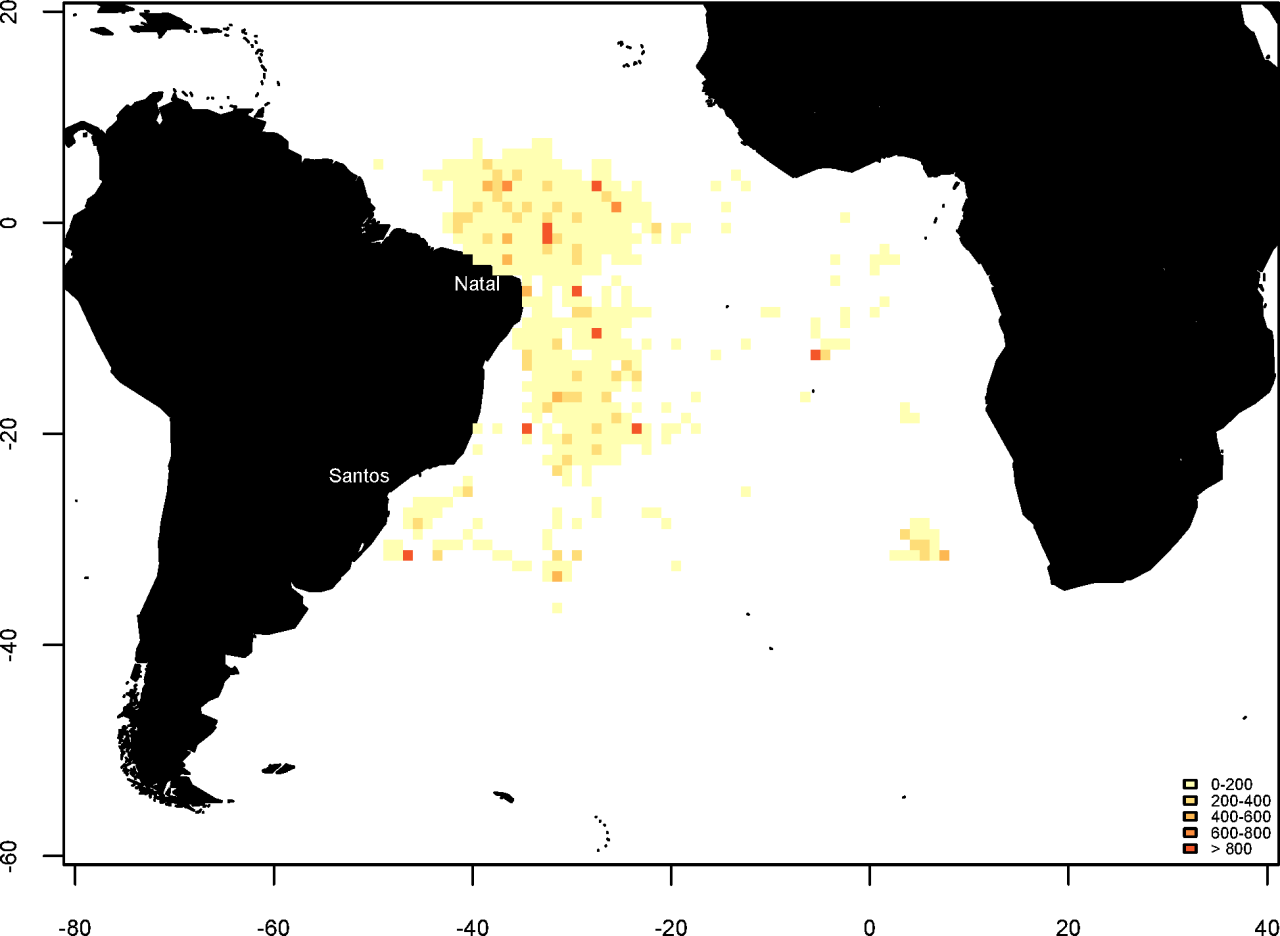
Sampling area showing geographic range and distribution of shortfin mako (*Isurus oxyrinchus*) caught by Brazilian chartered longline fleet between 2005 and 2011. Color range signifies sum of shortfin mako sharks caught by Brazilian chartered boats in the area covered by pixels of 1 x 1.

Shortfin mako sharks were sampled by onboard observers of the Brazilian pelagic longline fleet chartered from Spain, Panamá, Honduras, Morocco, Portugal and United Kingdom and based in northeast Brazil from 2005 to 2011 (Fig 1). Fork length (tip of the jaw to the center of the tail indentation, following the body curve) and sex, together with date of capture and geographical coordinates (latitude and longitude) were recorded. A block of five to seven vertebrae was removed from a subsample of individuals by onboard observers. It should be stated that the observers reported relative difficulty in collecting the biological material from the species due to the imposition on the part of the boat captains to preserve the carcasses for commercial purposes. Length mentioned hereafter refers to fork length (FL, cm). Histograms (10-cm classes) were used to assess the size structure of males and females (for both the total sample and subsample of vertebrae). Size frequencies of both sexes were compared using the two-sample Kolmogorov-Smirnov test. The sex ratio was analyzed using the χ^2^-test. The level of significance for all statistical tests was 5% (*p*-value ≤ 0.05).

### Age and growth

Vertebrae were cleaned and fixed in 4% formaldehyde for 24 h and stored in 70% alcohol. Each vertebra was embedded in polyester resin and sectioned to a thickness of approximately 0.3 mm using a low speed metallographic saw [38–40]. Growth BPs consisting of one wide band (opaque) and one narrow band (translucent) were counted and measured with the aid of a stereomicroscope at a magnification of 10x [38–40]. Only transmitted light was used so that narrow bands would appear translucent and wide bands would appear opaque. The Image Pro-plus software was used as an auxiliary tool. Distances (mm) from the focus of the vertebra to the outer margin of each *n*^*th*^ BP (*d*_*n*_) and to the edge of the section (vertebral radius - *VR*) across the corpus calcareum were recorded [38–40].

All sections were read twice by the same reader at different times (2011 and 2014) without previous knowledge of individual information, such as sex or size. The first reading in 2011 was used as reference. To ensure that the reading criteria were the same between the two periods, images of slides with clear views of the BPs were taken of the reference reading and used as the known reading set. The average percentage error (APE) and the average coefficient of variation (ACV) [38–42]were used to estimate the precision of growth band counts between readings using the following equations:

$$APE=100\%\times \frac{1}{R}\sum_{i=1}^{R}\frac{\left|X_{ij}-X_{j}\right|}{X_{j}}$$

$$CV=100\%\times \frac{\sqrt{\sum_{i=1}^{R}\frac{{\left(X_{ij}-X_{j}\right)}^{2}}{R-1}}}{X_{j}}$$

in which *R* is the number of readings of individual *j*; *X*_*ij*_ is the age of individual *j* as estimated by the *i*^*th*^ reader; and *X*_*j*_ is the mean age calculated for individual *j*. A bias plot was additionally constructed to verify the consistency between readings. Whenever the APE and/or CV was greater than 10%, a third reading was performed using the Image Pro-plus software. If no consensus was reached, the vertebrae in question were discarded. To test whether males and females have different growth dynamics, linear regressions were performed using logarithmized scales to extract coefficients of proportionality between structure (vertebra radius, VR) and size (FL), followed by testing with ANCOVA.

The monthly marginal increment ratio (MIR) was analyzed to determine the periodicity of BP deposition as well as to identify the period in which the narrow band is formed and a new BP begins [43]. The MIR was analyzed using the entire sample as well as subdividing the sample based on sex and age class (≤ 5 BPs and ≥ 6 BPs). Newborns with only the birthmark (BM) on the vertebrae were not considered in the MIR analyses. The following equation was used: *MIR* = (*VR* − *R*_*n*_)/(*R*_*n*_ − *R*_*n*−1_); in which *R*_*n*_ is the distance from the core to the end of the last complete BP and *R*_*n*−1_ is the distance to the end of the penultimate complete BP. Monthly mean and standard deviation (± *SD*) values were analyzed using the Kruskal-Wallis test [44].

Most fish stock assessment models rely only on von Bertalanffy growth function (*VBGF*) estimates [45]. In the present study, the decision was made to use only the traditional form of the *VBGF* [46, 47], as recently recommended [45]: $L_{t}=L_{inf}\left\{1-\;e^{-k\left(t-t_{0}\right)}\right\}$, in which *L*_*t*_ is length at age *t*, *L*_*∞*_ is asymptotic length, *k* is the growth coefficient and *t*_*0*_ is the theoretical age at which the individual has zero length. This formula was used to calculate growth parameters for males and females under three different scenarios: s1) considering an annual BP formation pattern; s2) considering the formation of two BPs per year; and s3) considering the formation of two BPs per year only in the first five years of life. The statistical performance of the models adjusted to the different hypotheses was evaluated using the Akaike information criterion (AIC) [48]. Kimura’s likelihood test was then used to compare growth parameters (isolated and combined) for males and females [49].

Age-length keys (ALKs) were used to identify the age composition for the entire sample [50–52]. Length class intervals were fixed at 10 cm (FL) and contingency tables were used to plot the frequency (%) of individuals from specific age classes in specific length classes [50–52]. Age at maturity (Tmat) was converted through the use of ALKs considering the sizes at 50% maturity for males and females reported by Natanson et al. (2006) for the North Atlantic, as this was the closest study to the area analyzed in the present investigation. Longevity (*ω*) was considered to be the age at which 99% of the theoretical maximum size is reached and estimated using the Fabens algorithm (7.21*ln*2/*k*), which is the most appropriate model for the estimation of longevity among elasmobranchs [38–40]. Considering the age composition for the entire sample and geographical coordinates in which the individuals were caught, maps were plotted to determine the spatial variability in life stages. Due to the numerous uncertainties regarding the biological parameters of the shortfin mako, the decision was made to divide the lifespan into three main classes: N = newborns or young of the year—YOY (0 BPs), Y = youngs (> 0 and < 6 BPs) and A = adults and sub-adults (> 6 BPs).

## Results

**Fig 2:**
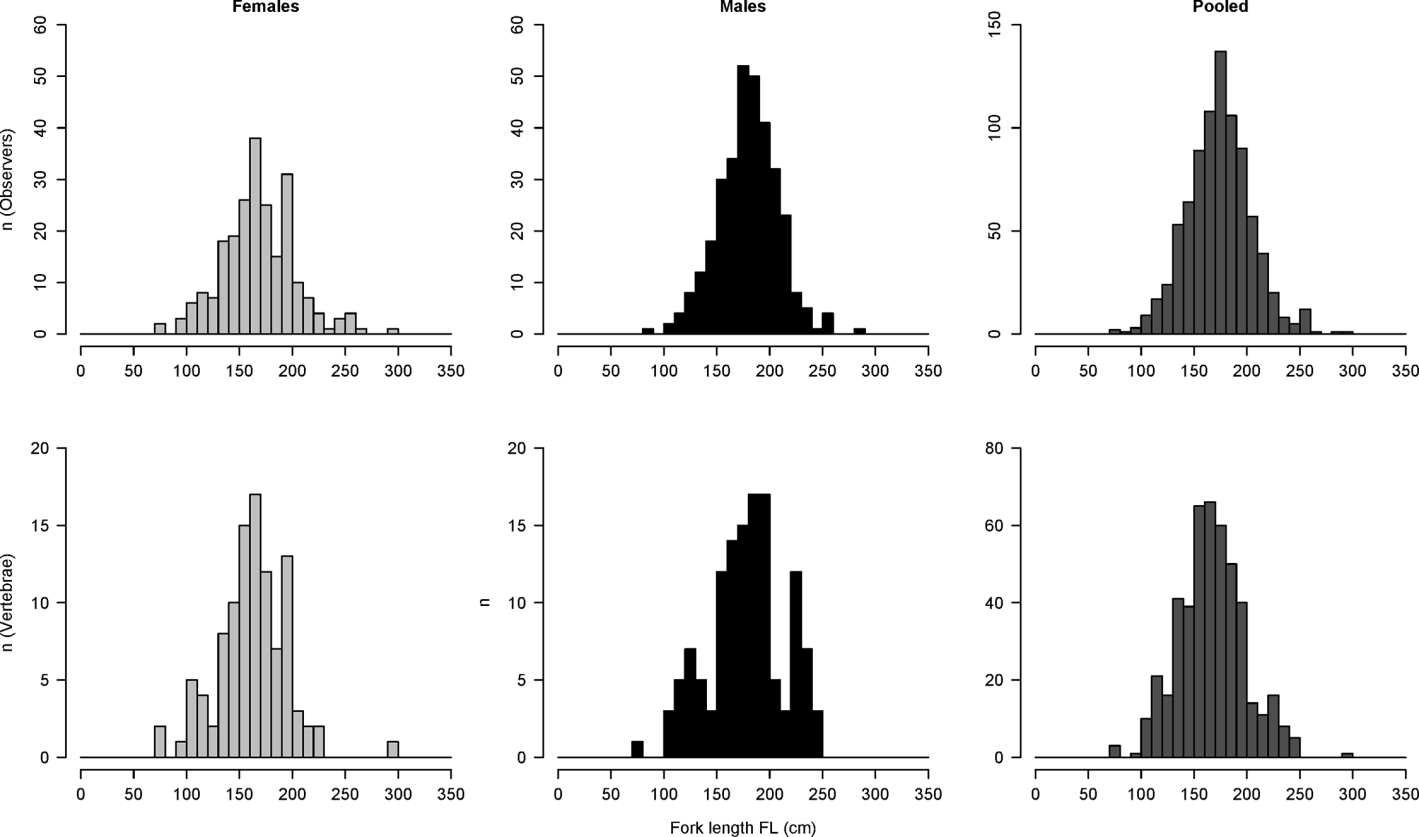
Length frequency distribution of shortfin mako sharks (*Isurus oxyrinchus*) sampled by onboard observers on Brazilian chartered fleet from 2005 to 2011.

A total of 1,325 individuals (385 females, 498 males and 442 of undetermined sex) were recorded (Fig 2, top). The ratio of males to females (1.29:1) was significantly different from 1:1 (X^2^ = 14.2061, p = 0.0001638) indicating that males are predominant in the region. Size was determined for 824 individuals and ranged from 76 to 296 cm. The Kolmogorov-Smirnov test indicated significant differences in the length distribution between sexes (*D* = 0.2464, p-value = 2.464e-07). The onboard observers encountered difficulties collecting the data: Sizes, sexes, geographic coordinates of the gear deployments and vertebrae were not collected in a consistent manner, which resulted in a large amount of useless information (approximately two thirds of the data collected).

**Fig 3:**
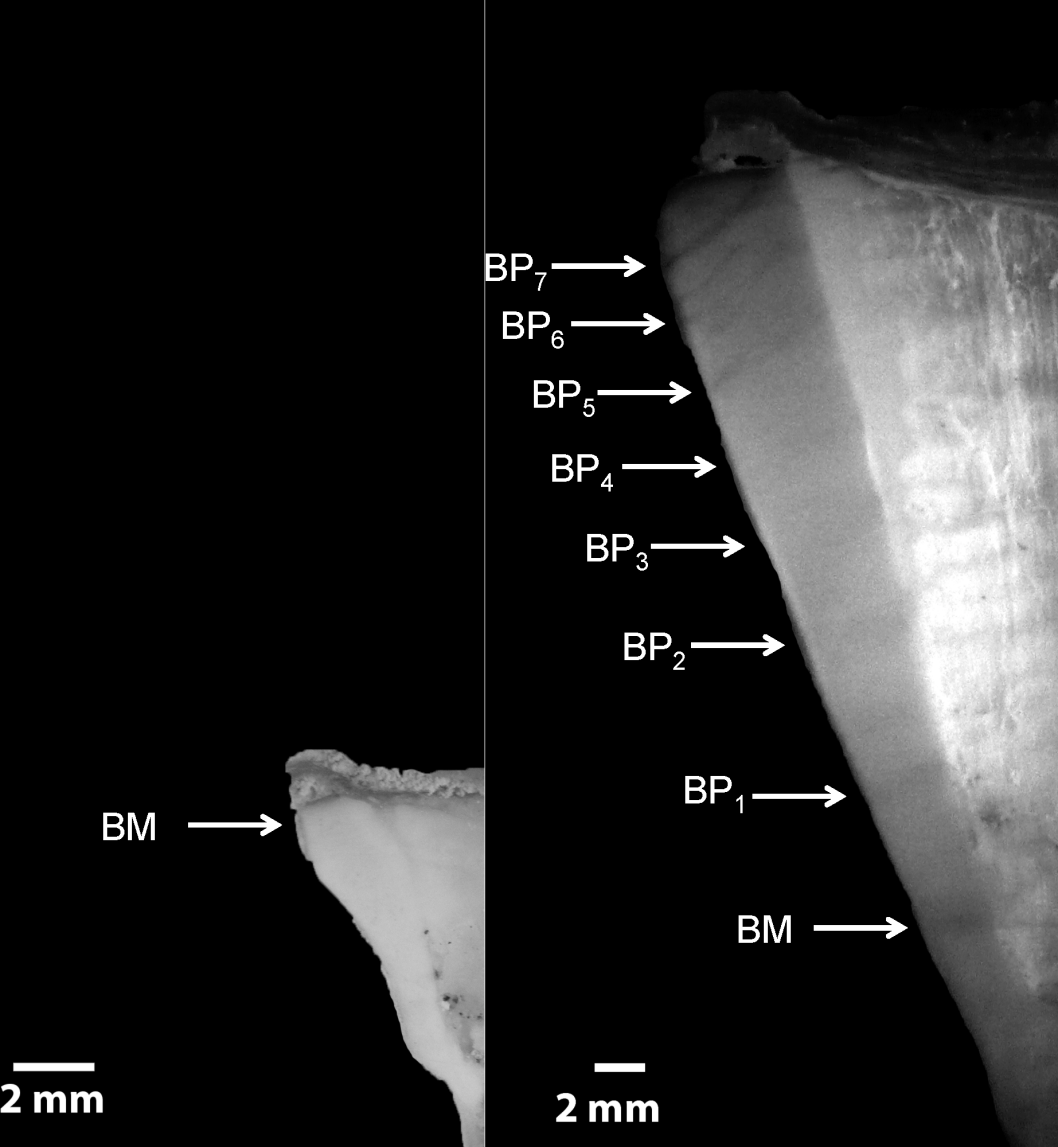
Images of vertebrae sections from two individuals showing band pairs. A = Female with 2 BPs; B = Male with 9 BPs.

**Fig 4:**
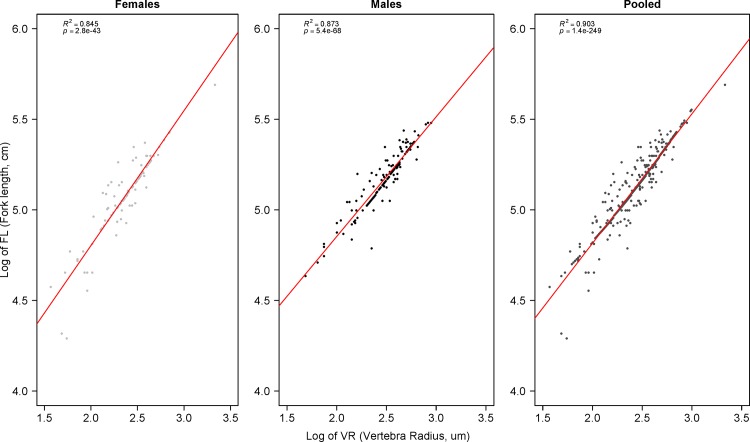
Log-transformed fork length (FL) vs. vertebra radius (VR) in shortfin mako (*Isurus oxyrinchus*) sharks caught by Brazilian chartered longline fleet and reported by onboard observers from 2005 to 2011.

A total of 467 vertebrae from 129 males, 104 females and 234 individuals of undetermined sex were analyzed to estimate growth parameters (Fig 2, bottom). The first distal narrow band to the focus was interpreted as the birthmark (BM), which is equivalent to age 0+ (Fig 3). The relationship between FL and VR was slightly curvilinear (particularly for males), indicating allometric growth. One cannot discard the hypothesis that this effect may have been caused by the considerable size of the area sampled. Thus, data were log transformed to allow linear regression (Fig 4). Significant effects of both VR and sex were found on FL, but no significant interaction was determined (as calculated using ANCOVA), which suggests that the regression slopes between VR and FL were similar for both sexes. However, sex had a significant effect on FL, which can be interpreted as a significant difference between intercepts on the regression lines for males and females. Therefore, the decision was made to present the findings separately for the different sexes.

**Fig 5:**
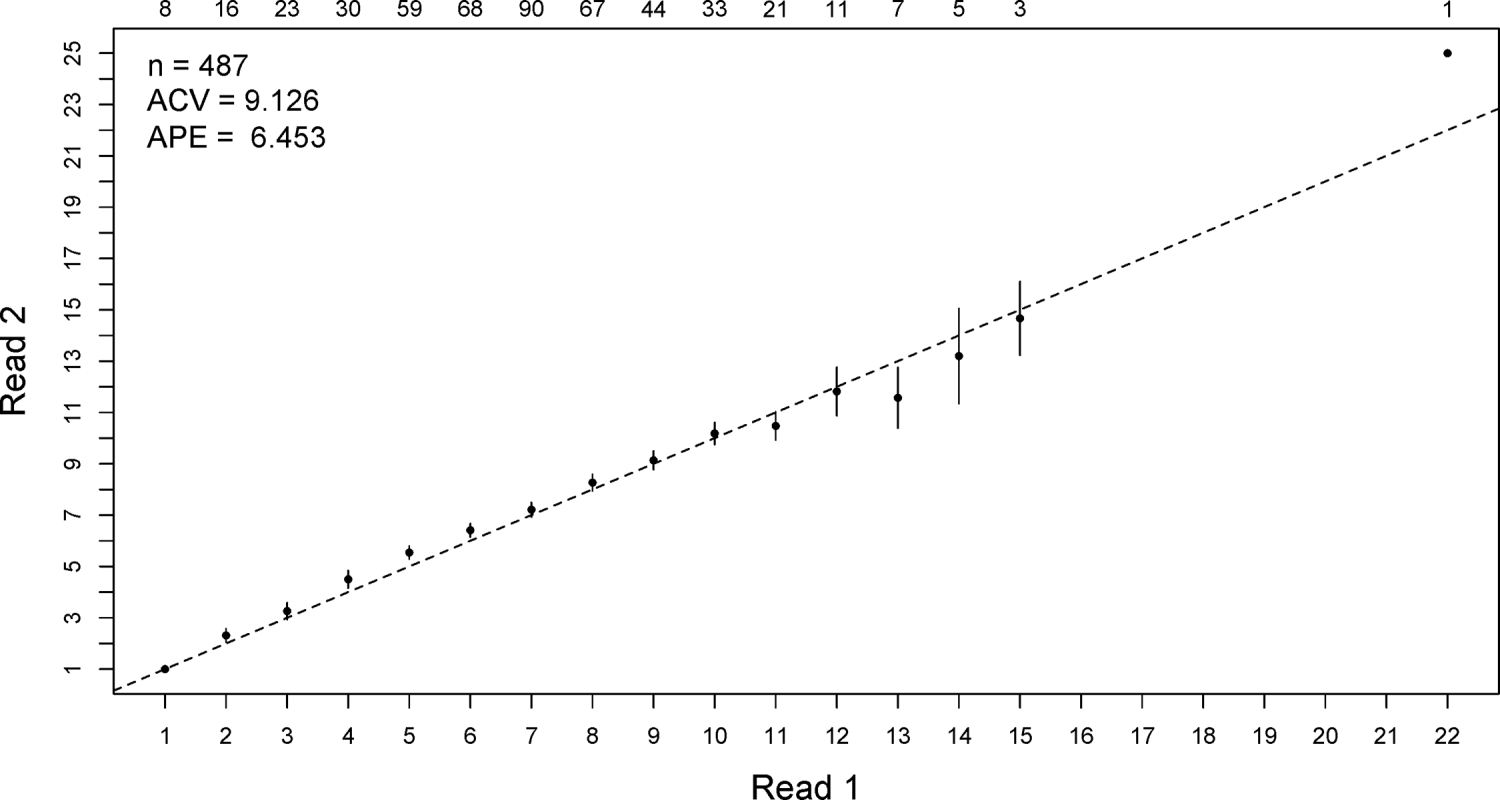
Age bias plot of shortfin mako (*Isurus oxyrinchus*) caught by Brazilian chartered longline fleet.

**Fig 6:**
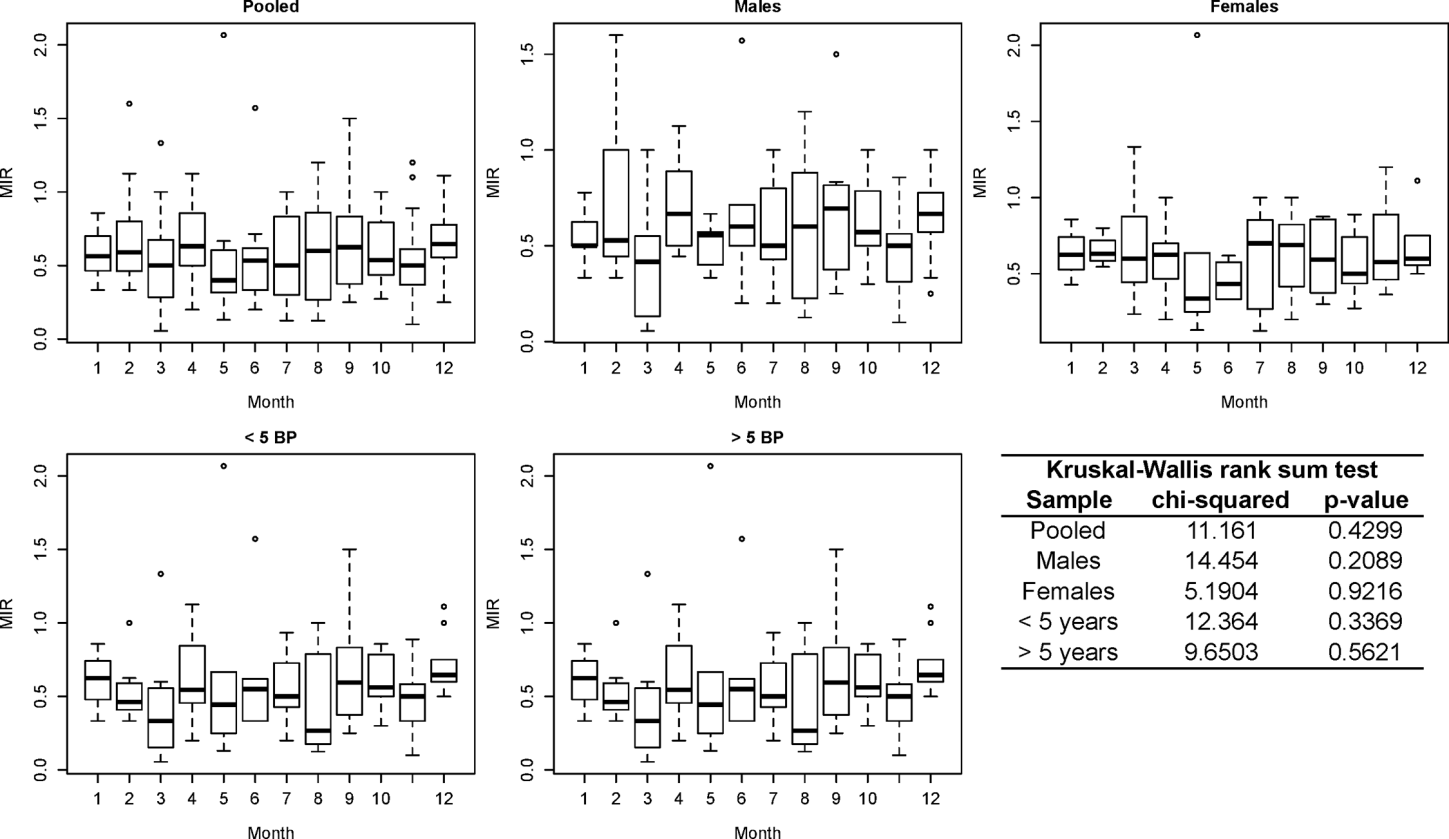
Median vertebral marginal increment ratio (MIR) and Kruskal-Wallis rank sum test outputs performed for different subsamples of shortfin mako (*Isurus oxyrinchus*) caught by Brazilian chartered longline fleet between 2005 and 2011 (n = 448).

The APE and ACV between readings were (respectively): 6.45 and 9.12% (Fig 5). The rate of prefect agreement regarding age assignments based on vertebrae was approximately 35%. However, approximately 25% of the slides had an APE greater than 10% and were read again (new BP count) using the Image Pro-plus 7 software, which resulted in a reduction in the total APE on the order of 4.5-fold (6.5 to 1.4). No vertebra was discarded. No significant differences were found among monthly medians of the MIR calculated for the different scenarios (Fig 6). In a merely subjective interpretation, the smallest MIRs observed in all scenarios were found in May.

Since the MIR analyses were inconclusive, the decision was made to consider the results of all three BP deposition period hypotheses to interpret ages and fit VBGF growth curves. Observed ages (considering 1 BP deposition per year–s1) ranged from 0 to 23 years for females (76 to 296 cm FL, respectively) and 0 to 11 years for males (79 to 250 cm FL, respectively). Considering the pattern of 2 BP depositions per year (s2), observed ages ranged from 0 to 11 years for females and 0 to 5 years for males. Finally, using the hypothesis of 2 BPs per year until five years of life (s3), ages ranged from 0 to 19 years for females and 0 to 6 years for males. The sample was characterized by an absence of individuals larger than 200 cm (FL).

**Fig 7:**
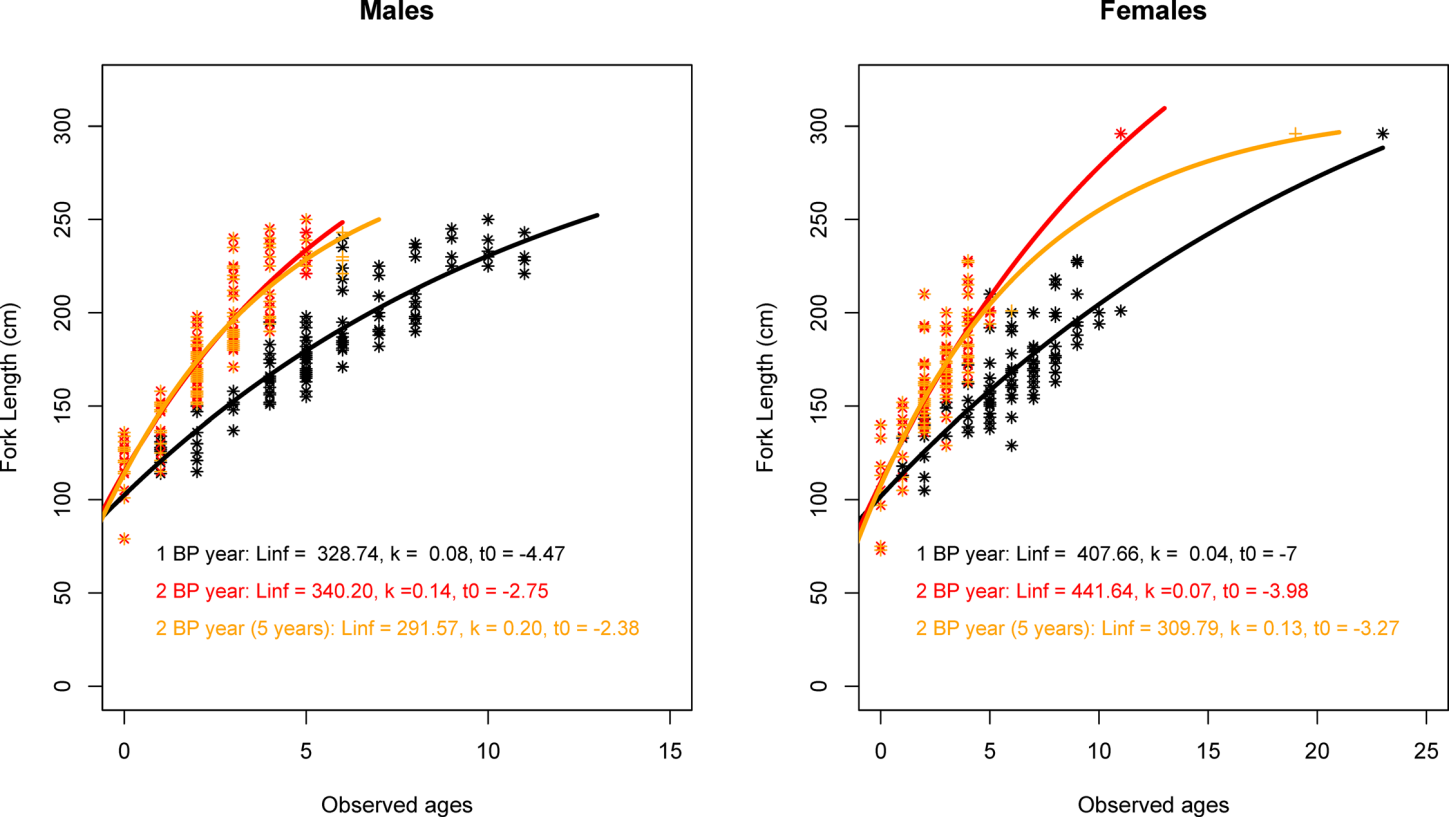
Observed ages, growth curves and estimated parameters for shortfin mako (*Isurus oxyrinchus*) caught by Brazilian chartered longline fleet between 2005 and 2011. Black dots (and line) represent observed ages, growth curves and growth parameters estimated considering one band pair deposition annually. Red dots (and line) represent observed ages, growth curves and growth parameters estimated considering deposition of two band pairs annually. Orange dots (and line) represent observed ages, growth curves and growth parameters estimated considering deposition of two band pairs annually until five years of life, followed thereafter by one BP annually.

Table 1 and Fig 7 show the growth parameters estimates of the *VBGF* models and fitted curves for males and females in the three different scenarios (s1-s3). Males exhibited higher growth rates (k) and smaller maximum asymptotic lengths than females in all situations. Kimura`s likelihood ratio tests indicated statistically significant differences between the growth curves of males and females in all scenarios used. The same test also indicated statistically significant differences in growth curves for the same sex, specifically when hypotheses validated in the literature using high-precision methods (1 BP vs. 2 BPs until five years of life) were compared [32, 33, 37]. Statistically, the results indicate that the VBGF models had the best fit to observed ages when the pattern of 1 BP per year was considered (Table 2). Thus, based on these results, we assume annual periodicity and all further figures and tables are based on this assumption unless otherwise specified.

**Table 1:** Growth parameters (L_inf_, k and t0 with standard error [S.E] in parenthesis), Akaike information criterion (AIC) values, Akaike differences (*∆*_*i*_) and Akaike weights (*w*_*i*_) estimated for shortfin mako (*Isurus oxyrinchus*) using von Bertalanffy growth function (VBGF) and different scenarios of band pair deposition.

Sex	Scenario	*L*_*∞*_ (cm)	*k* (year^-1^)	*t*_*0*_ (years)	*AIC*	*∆*_*i*_	*w*_*i*_ (%)
Males	s1	328.74 (40.84)	0.08 (0.02)	-4.47 (0.73)	1053.38	0	100
	s2	340.20 (54.42)	0.14 (0.05)	-2.75 (0.47)	1075.03	21.63	0
	s3	291.57 (25.16)	0.20 (0.04)	-2.38 (0.35)	1079.22	25.82	0
Females	s1	407.66 (97.68)	0.04 (0.01)	-7.00 (1.32)	888.59	0	98.76
	s2	441.64 (136.29)	0.07 (0.03)	-3.98 (0.77)	898.79	10.2	0.6
	s3	309.79 (26.84)	0.13 (0.02)	-3.27 (0.47)	898.66	10.07	0.64

**Table 2:** Likelihood ratio tests (Kimura, 1980) for growth parameters estimated for shortfin mako (*Isurus oxyrinchus*) males and females caught by Brazilian chartered longline fleet (from 2005 to 2011) using von Bertalanffy growth function (VBGF) and different scenarios of band pair deposition.

Scenario	Test	Hypothesis	*χ*^*2*^	*p*
s1	Ho vs H1	Linf (M) = Linf (F)	1.66	0.198
	Ho vs H2	K (M) = K (F)	2.88	0.09
	**Ho vs H3**	**t0 (M) = t0 (F)**	**3.86**	**0.049**
	**Ho vs H4**	**M (all) = F (all)**	**31.63**	**0**
s2	Ho vs H1	Linf (M) = Linf (F)	1.5	0.221
	Ho vs H2	K (M) = K (F)	2.19	0.139
	Ho vs H3	t0 (M) = t0 (F)	2.89	0.089
	**Ho vs H4**	**M (all) = F (all)**	**27.12**	**0**
s3	Ho vs H1	Linf (M) = Linf (F)	1.5	0.221
	**Ho vs H2**	**K (M) = K (F)**	**3.83**	**0.05**
	**Ho vs H3**	**t0 (M) = t0 (F)**	**4.17**	**0.041**
	**Ho vs H4**	**M (all) = F (all)**	**24.29**	**0**

**Fig 8:**
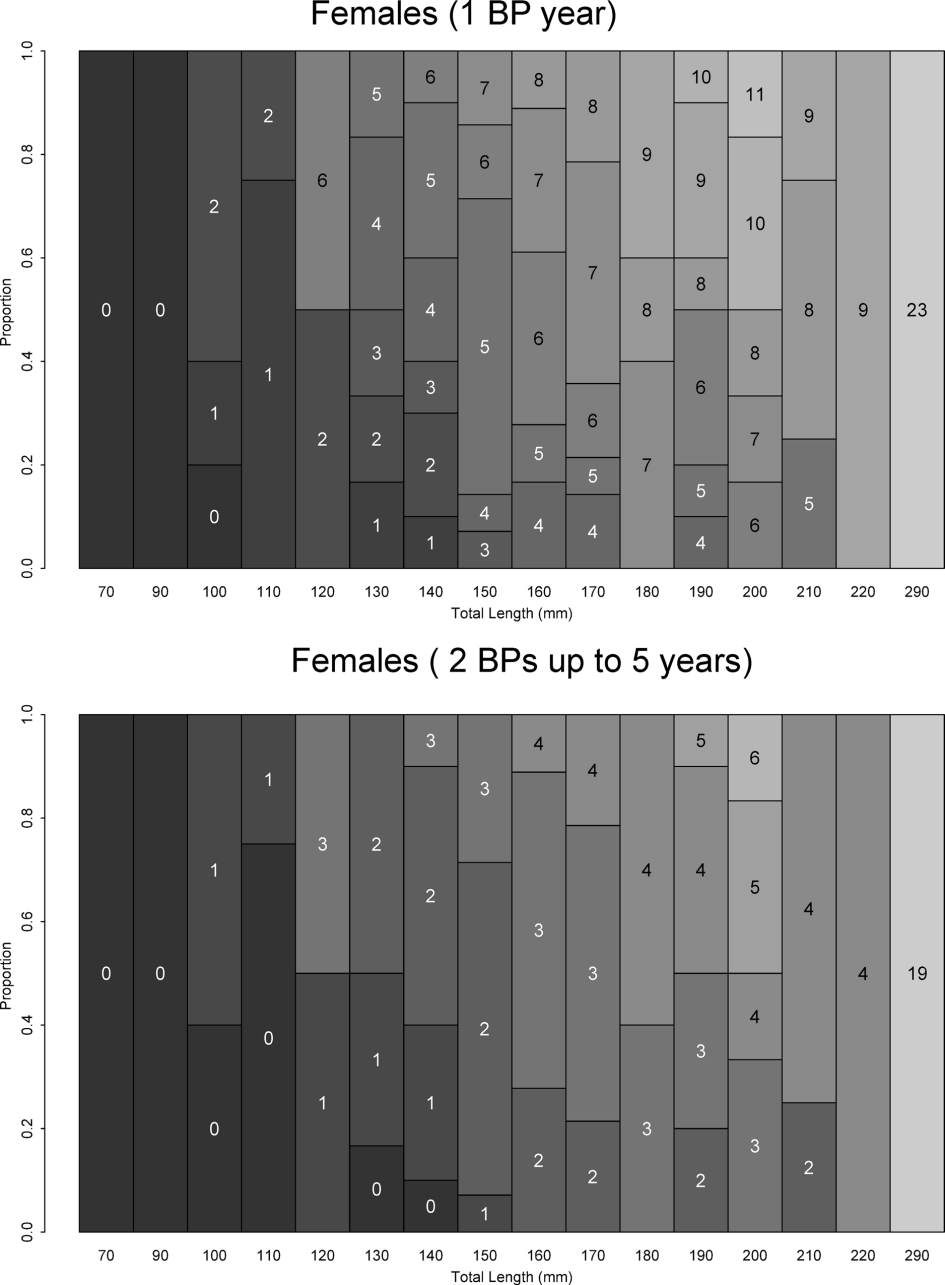
Age-length key (ALK) for shortfin mako (*Isurus oxyrinchus*) females caught by Brazilian chartered longline fleet from 2005 to 2011 considering fully validated hypotheses of band pair (BP) deposition (Campana et al. 2002; Natanson et al. 2006; Wells et al. 2013).

**Fig 9:**
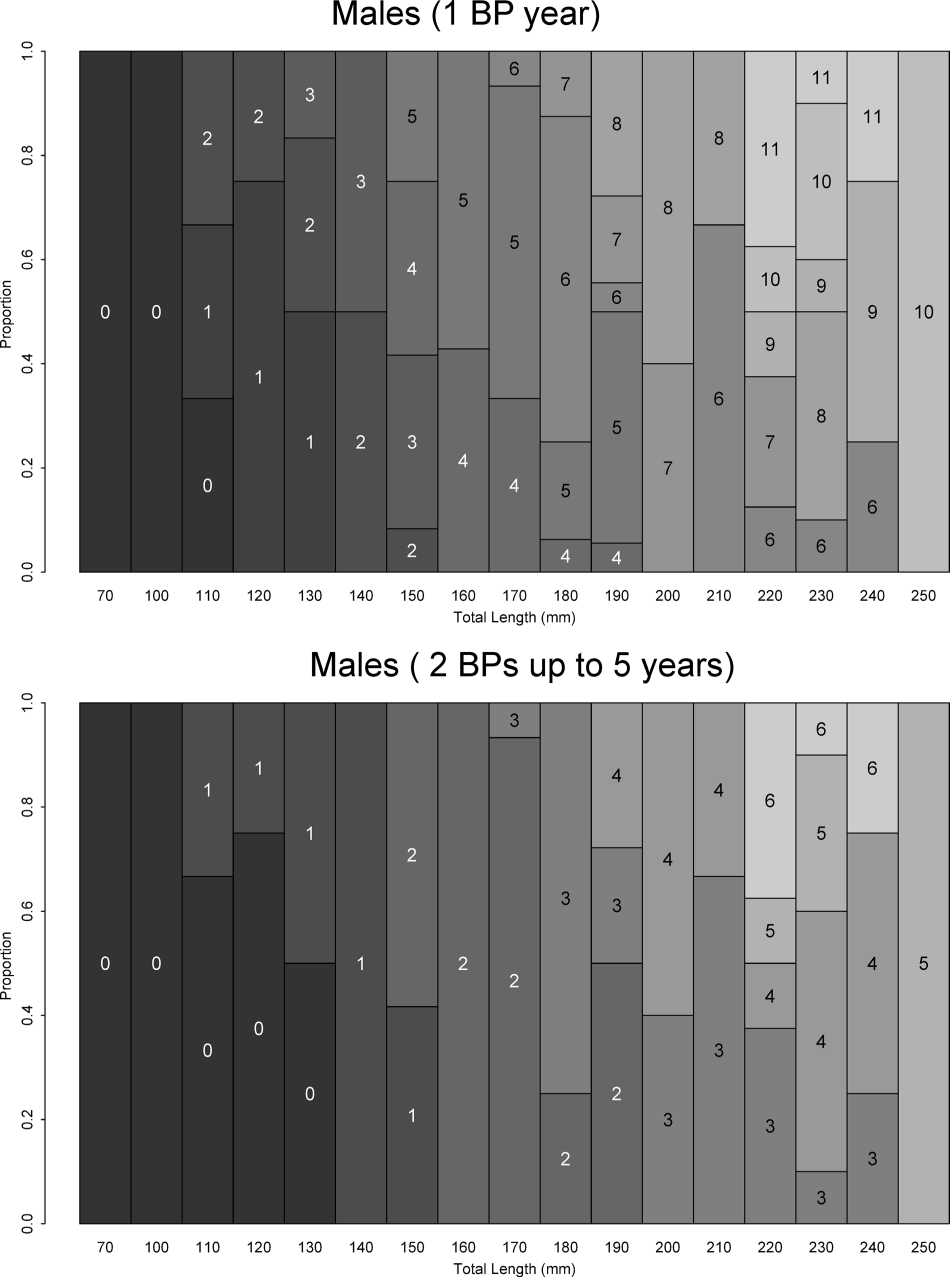
Age-length key (ALK) for shortfin mako (*Isurus oxyrinchus*) males caught by Brazilian chartered longline fleet from 2005 to 2011 considering fully validated hypotheses of band pair (BP) deposition (Campana et al. 2002; Natanson et al. 2006; Wells et al. 2013).

**Fig 10:**
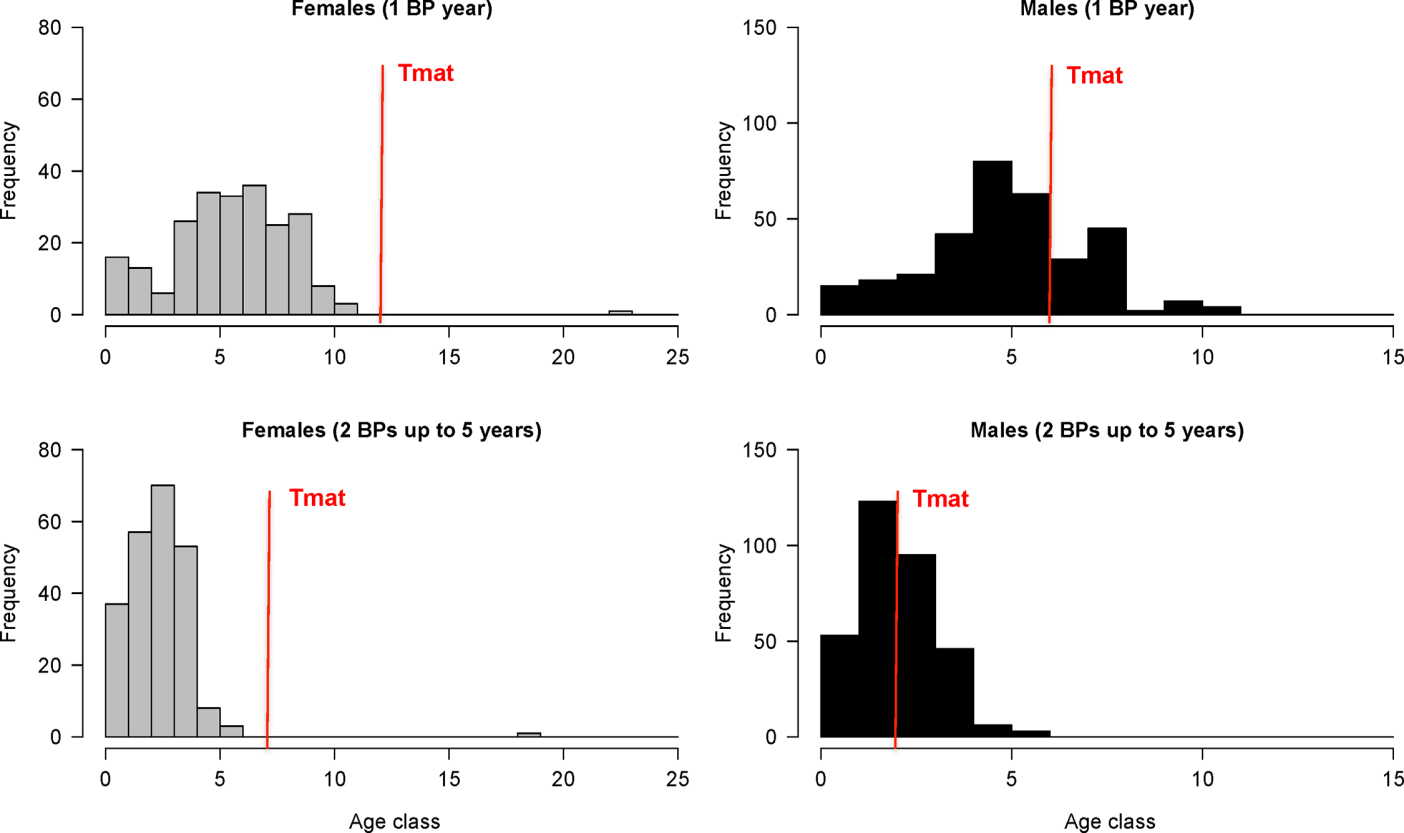
Age composition (entire sample) of shortfin mako (*Isurus oxyrinchus*) caught by Brazilian chartered longline fleet and reported by onboard observers from 2005 to 2011.

AKLs are offered in Figs 8 and 9 for females and males using BP scenarios 1 and 3 (s1 and s3). In both situations, observed age structure in each length class was more homogenous (fewer variations) for males than females. For scenarios s1-s3, age at first maturity ranged from 7 to >12 years for females and 3 to 6 years for males. Estimated longevity ranged from 19 to 28 years for females and 16 to 23 years for males. The frequency distribution by age class demonstrates that most fish were less than 10 years of age (Fig 10). Only one adult female was observed.

**Fig 11:**
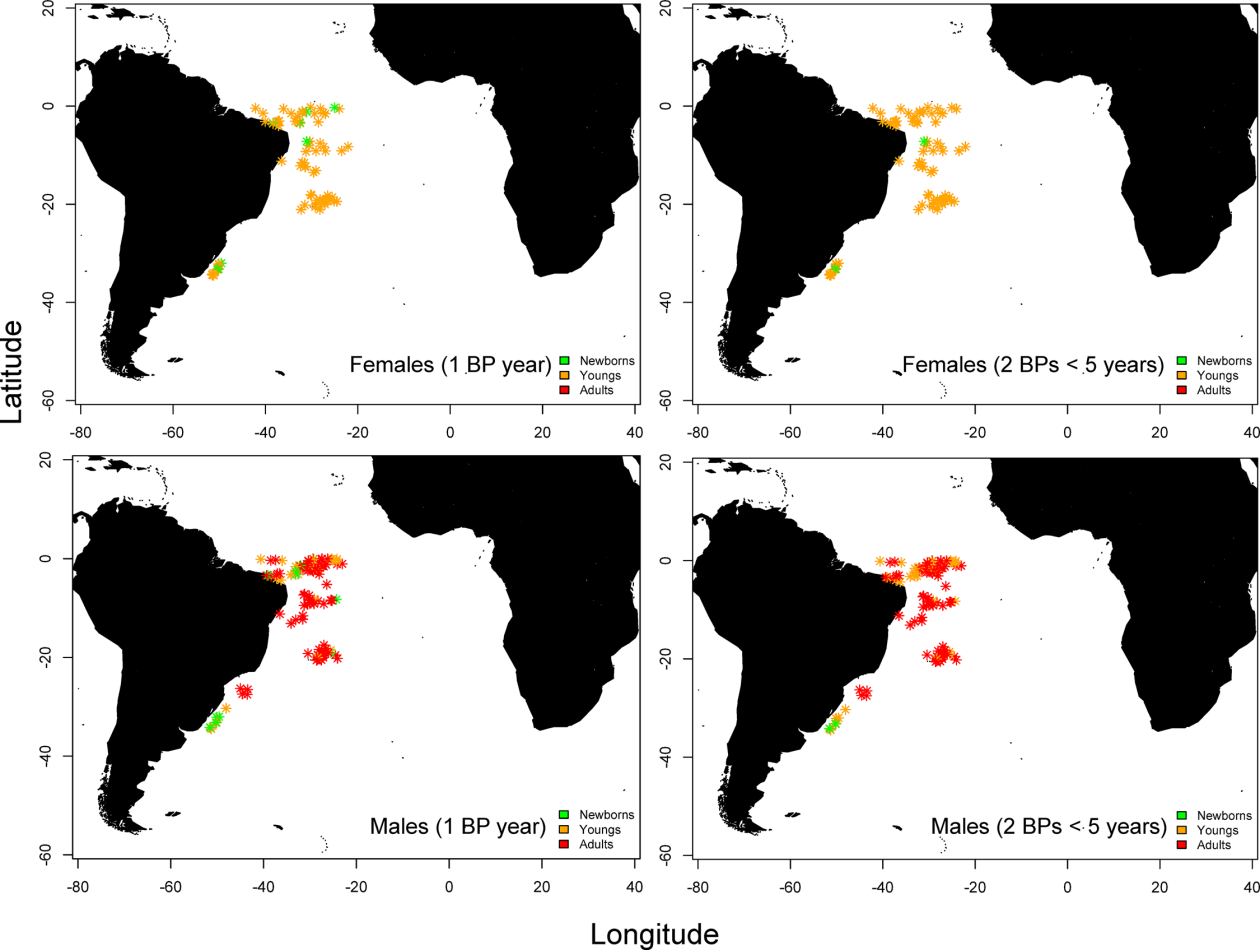
Spatial distribution of shortfin mako (*Isurus oxyrinchus*) caught by Brazilian chartered fleet between 2005 and 2011 considering fully validated hypotheses of band pair (BP) deposition (Campana et al. 2002; Natanson et al. 2006; Wells et al. 2013). Green = young of the year (YOY) or newborns; Orange = Young individuals between ages 1 and 5; Red = Sub-adults and adults (> *Tmat*).

Regarding the spatial distribution of the lifecycle phases, adult females were rare (n = 1, not included in the map due to the unavailability of coordinates of the set for this individual), whereas males in all phases were found in practically the entire sample (Fig 11). Individuals classified as newborns, particularly males, were exclusively found in the region from southern Brazil to Uruguay. Unfortunately, many individuals (n = 1056) were not considered for spatial analysis because biometry and coordinates were not recorded.

## Discussion

The present findings indicate that shortfin mako sharks exploited by the Brazilian chartered fleet in the western and central South Atlantic are predominantly immature, especially females. The size structure for both males and females was similar to that reported in previous studies on the species (Table 3). Individuals greater than 200 cm (FL) were extremely rare for both sexes, which is in agreement with data described in the literature and may be associated to both gear selectivity as well as ecological aspects, such as habitat preference (spatial and/or seasonal segregation) or overfishing, but one cannot discard the highly migratory characteristic of this species [2, 7, 22, 37].

**Table 3:** Growth studies conducted with shortfin mako by authors in different locations [updated from Natanson et al. (2006)].

Reference	Sex	FL (cm)	Linf	k	t0	n	Área	Tmat	Tmax	BP (year)	Longevity
Pratt and Casey (1983)	M	69–238	302	0.26	-1	49	Western NA	3	4.5	2	10
	F	69–238	345	0.2	-1	54		7	11.5	2	14
Cailliet and Bedford (1983)	M	80.6–293	298	0.07	-3.75	44	Pacific, California	7	17	1	38
	F	80.6–293	298	0.07	-3.75	44					
Chan (2001)	M	66–274	267	0.31	-0.95	24	Pacific, Australia	-	7	2	9
	F	74–314	349	0.15	-1.97	52		-	10	2	17
Hsu (2003)	M	72.6–250.9	321.8	0.04	-6.07	133	China	13	23.6	1	3
	F	72.6–314.9	403.62	0.04	-5.27	174		18	30.6	1	-
Ribot-Carballal et al. (2005)	M	68.6–264	375.4	0.05	-4.7	109	Pacific, Baja	7	18	1	55
	F	68.6–264	375.4	0.05	-4.7	109		15			
Bishop et al. (2006)	M	100–347	302.2	0.05	-9.04	145	Pacific, New Zealand	8	29	1	48
	F	-	820.1	0.01	-11.3	111		20	28	1	219
Natanson et al. (2006)**	M	72–260	253.3	0.12	L0	118	Western NA	8	29	1	21
	F	64–340	365.6	0.08	L0	140		18	32	1	38
Cerna and Lincandeo (2009)	M	70–258*	268.07*	0.08	-3.58	243	Pacific, Chile	-	25	1	-
	F	69–300*	295.73*	0.07	-3.18	304		-	25	1	-
Semba et al. (2009)	M	73–265*	255*	0.16	L0	128	Western and central NP	8	14	1	-
	F	73–330*	340*	0.09	L0	147		20	19	1	-
Doño et al. (2014)***	M	81–250	416	0.03	-6.18	116	Southwest SA	8	18	1	-
	F	101–330	580	0.02	-7.52	126		18	28	1	-
This study	M	79–250	328.74	0.08	-4.47	129	West and Central SA	6	11	1	23
	F	73–296	407.65	0.04	-7.08	109		>12	23	1	28
	M	79–250	340.2	0.14	-2.75	129		3	6	2	19
	F	73–296	441.64	0.07	-3.98	109		6	11	2	24
	M	79–250	291.57	0.2	-2.38	129		3	6	2****	16
	F	73–296	309.79	0.13	-3.27	109		>7	19	2****	19

*FLs were estimated from study-specific conversion equations;

** Use of Gompertz GF;

*** Paramters from VBGF (authors reported Schnute`s GF best fit);

**** 2 BPs up to the first five years of life

The size structure differed between sexes both with regard to the proportion of individuals in each class and the proportionality between the structure (vertebra) and length of the individuals, evidencing sexual dimorphism regarding both size selectivity and individual growth. These differences were expected, as the literature reports sexual dimorphism for this species, with males reaching maximum sizes of around 260 cm (FL) and females reaching around 340 cm (Table 3).

An important aspect of the present database was the considerable amount of individuals with no information on size or even sex, which implies in discarding of a large amount of data (approximately 35% of information on size and 50% on vertebrae). Nonetheless, the vertebrae proved useful for ageing, as demonstrated by the high degree of reproducibility between readings. Despite the small number of older sharks, the authors believe that the sample was sufficient for the estimation of growth parameters for the shortfin mako, given that individual BP counts related to size were very similar to those reported in previous studies (Table 3). For example, Wells et al. (2013) observed sizes close to or less than 200 cm and vertebrae with a maximum of 12 BPs. A large part of the present sample was composed of individuals with a maximum of 200 cm (FL) and 15 BPs; only one female had more than 15 BPs (Fig 7).

Given the inability to validate periodicity in BP deposition (which was conducted using only an indirect method) and the need to suggest an age band formation pattern for the species, the BPs in the present study demonstrate a better statistical performance when adjusted to the growth model considering an annual deposition of BPs. However, three hypotheses regarding the BP deposition pattern were tested (s1,s2 and s3). Despite this better performance for the annual pattern, AIC values were relatively close, but the same cannot be said with regard to growth parameters (Table 3).

Researchers have debated the age band deposition pattern in the shortfin mako for decades [27–37]. According to Bishop et al. 2006 and Wells et al. 2013, the estimates and conclusions reported by all authors cited (Table 3) differ fundamentally with regard to the deposition pattern used to interpret BP deposition. The same authors also report that, when the same deposition pattern is used, the curves are similarly adjusted, thereby providing relatively close growth patterns, which indicate that the methods employed by different research groups produce similar results [21, 37].

This was found between the present investigation and the reference study for the South Atlantic [36]. Both size structures and growth curves were similar, but estimated growth parameters (both considering the annual BP deposition pattern) using the VBGF were dissimilar (Table 3). The authors cited suggest better statistical support (and other parameters that have a differentiated interpretation in relation to those estimated using the VBGF) when the Schnute model was used in a Bayesian approach [36].

Wells et al. (2013) recently validated a deposition pattern of 2 BPs per year using OTC-marked vertebrae for 29 individuals up to five years of age. As previously reported by Natanson et al. 2006, the authors reviewed all studies and their interpretations and found that, besides the differences in the BP deposition pattern and the low frequency of large or older individuals, nearly all authors stated that the shortfin mako likely exhibits ontogenetic variation in its growth rates. Such observations included studies that validated annual BP deposition, particularly Ardizzone et al. 2006 and Natanson et al. 2006, whose also report higher growth rates for young tag-and-recapture specimens in comparison to those estimated using vertebrae.

When the hypothesis suggested by Wells et al. (2013) is considered (two BPs until five years of life, followed thereafter by 1 BP annually–s3), the estimated growth parameters differ significantly from those estimated considering 1 BP (s1) per year (Fig 7) which is attributed to the consideration of more accelerated growth (BP depositions) in the early years of life [27]. While it is not possible to prove the deposition pattern in the South Atlantic and there is no tag-and-recapture information, the sensitivity of the present growth parameter estimates was tested, particularly for females (which included the oldest individual in the entire sample) using the VBGF, annual BP deposition and excluding the only older female. The exclusion of this adult female resulted in substantially different (and even more realistic) parameters (Linf = 260.88 [47.46 S.E]; k = 0.09 [47.46 S.E] and t0 = -4.74 [1.38 S.E]) and the AIC diminished considerably (879.33). Thus, our results indicates that shortfin mako sharks caught in the South Atlantic Ocean has more accelerated growth in the young phase of its life, as recently reported for populations in the North Atlantic and Pacific [32, 33, 37].

Regional, governmental and conservation management organizations of the South Atlantic should prioritize studies directed at clarifying the BP deposition pattern in the shortfin mako as well as its reproductive aspects. Such information is fundamental to accurate assessments. There is also an immediate need to gain a better understanding of habitat use and behavior for this species, as large mature females were rarely found in both studies conducted in the western South Atlantic [36]. As a last note, the authors of this paper are particularly concerned with the extinction of the Onboard Observer Program by the Brazilian government, which occurred in 2012, since this hinders the possibility of future studies on the bioecology of the shortfin mako as well as oceanic fishing operations targeting this species.
